# Degradation of the Nonsteroidal Anti-Inflammatory Drug Piroxicam by Iron Activated Persulfate: The Role of Water Matrix and Ultrasound Synergy

**DOI:** 10.3390/ijerph15112600

**Published:** 2018-11-21

**Authors:** Zacharias Frontistis

**Affiliations:** Department of Environmental Engineering, University of Western Macedonia, GR-50100 Kozani, Greece

**Keywords:** piroxicam, NSAIDS, iron persulfate, water matrix, ultrasound, Fenton like, sulfate radicals, water matrix, ultrasound, synergy

## Abstract

This work examined the oxidation of Piroxicam (PIR), a representative nonsteroidal anti-inflammatory drug using iron activated persulfate. The effect of persulfate dosing was vital for the efficiency of the process. The addition of 20 mg/L sodium persulfate (SPS) eliminated 500 μg/L of PIR in less than 20 min at natural pH. PIR decomposition followed pseudo-first-order kinetics, and the observed kinetic constant increased by 2.1 times when the initial concentration of PIR decreased from 2000 to 250 μg/L. Acidic pH favored the PIR destruction, while both sulfate and hydroxyl radicals are involved in PIR destruction at natural pH. The effect of inorganic ions like bicarbonate and chlorides was almost insignificant on PIR removal. The presence of humic acid reduced PIR removal from 100% to 67% after 20 min of treatment with 2 mg/L Fe^2+^ and 20 mg/L SPS. The experiment that was performed with bottled water showed similar efficiency with ultrapure water, while in the case of secondary effluent, PIR removal decreased by 26% after 30 min of treatment. The Fe^2+^/SPS/ultrasound hybrid process showed a low degree of synergy (18.3%). The ecotoxicity of aqueous solution using the *Vibrio fischeri* as an indicator was reduced during the treatment, although with a different trend from the removal of PIR, possibly due to byproducts derived from the oxidation of secondary effluent and PIR.

## 1. Introduction

Nowadays, there are several reports on the detection of pharmaceuticals in environmental samples such as surface water and groundwater, and even sediments [1,2,3]. Since most pharmaceuticals have been designed to be resistant to microorganisms, their removal from water is a major challenge [4]. Conventional biological systems are inadequate to remove these substances which are often called xenobiotics from urban wastewater [4,5,6]. A large class of pharmaceuticals is non-steroidal anti-inflammatory drugs (NSAIDs) [7]. This family includes drugs that are widely consumed for different diseases such as diclofenac (DCF), naproxen (NPR), ibuprofen (IBP) and piroxicam (PIR) [7,8].

There is a large number of studies on the removal of NSAIDs through physical processes such as adsorption with activated carbon and biomass-derived materials [9,10,11,12]. However, adsorption presents the disadvantage of saturation of the surface of the adsorbent and the need for regeneration, which is usually difficult or costly.

An interesting alternative is the implementation of a relatively new generation of technologies called Advanced Oxidation Processes (AOPs) [4,5]. These physicochemical processes are based on the in situ generation of very reactive oxygen species. AOPs include, among others, technologies like Ozonation, sonochemistry, photocatalysis, electrolysis and Fenton reaction [4]. However, one major disadvantage of AOPs that prevents their extensive use is that most processes such as ozone or electrochemical oxidation require very large amounts of energy or they suffer from mass transfer limitations [13].

On the other hand, Fenton reaction is a low-cost technology based on the production of reactive oxygen species according to the following reaction [14]:

(1) $${\mathrm{Fe}}^{\mathrm{II}}+H_{2}O_{2}\to {\mathrm{Fe}}^{\mathrm{III}}+{}^{•}{\mathrm{OH}+\mathrm{HO}}^{‒}\;$$

In recent years, most researchers have focused on the simultaneous use of Fenton reagents with ultraviolet or solar light, which can reduce iron (III) to iron (II), thus enhancing the efficiency of the process [15].

There are also several reports on the application of solar Fenton in pilot plants for the degradation of different micropollutants [16,17,18]. More recently, some researchers have also demonstrated the simultaneous disinfection or removal of antibiotic-resistant genres using solar Fenton [19,20].

One of the major problems of the Fenton reaction is that the required hydrogen peroxide is costly and relatively difficult to transport and use, as it is liquid and decomposes quickly. In recent years, research has constantly been increasing about the use of alternative oxidants like persulfate [21]. Persulfate can be activated with several ways like transition metals, heat, UV radiation, ultrasound, electrochemical oxidation, and biochars [21,22,23,24].

(2) $$S_{2}O_{8}^{2‒}+\mathrm{activator}\to {\mathrm{SO}}_{4}^{•‒}+({\mathrm{SO}}_{4}^{•‒}\;\mathrm{or}\;{\mathrm{SO}}_{4}^{2‒})\;$$

Persulfate also has several advantages in transport and storage, since it is solid and has a long lifetime [21].

More recently, Lianou and coworkers examined the sonochemical degradation of the NSAID piroxicam [25]. Lower-frequency ultrasound at 20 kHz was capable of degrading up to 960 μg/L of PIR in less than 30 min using 36 W/L ultrasound power density. Kouskouki and coworkers [26] studied the electrochemical oxidation of piroxicam on a Boron-Doped Diamond. The electrochemical oxidation was capable of removing piroxicam in short treatment times (i.e., less than 15 min for the removal of 245 μg/L of Piroxicam at 26.7 mA/cm^2^). However, electrolysis, in addition to the high amount of energy required, also requires the addition of salts in order to increase the conductivity.

In the present study, the destruction of the NSAID Piroxicam using iron activated persulfate was investigated. The work focus on the effect of the different operating parameters, namely oxidant, pH, initial concentration and water matrix. In addition, the potential synergy with the simultaneous use of low-frequency ultrasound was also investigated. As far as we know, this is the first study on the degradation of the piroxicam using sulfate-based processes.

## 2. Materials and Methods

### 2.1. Chemicals

Sigma Aldrich was the supplier for humic acid (CAS number 68131-04-4), Piroxicam >98% (CAS number 36322-90-4), phosphoric acid (CAS number7664-38-2) and tert-Butyl alcohol (CAS number 75-65-0); sodium persulfate (CAS number 7775-27-1) was purchased from Scharlau, while acetonitrile (CAS number 75-05-8) and methanol (CAS number 67-56-1) were purchased from Panreac. Some experiments were also performed using bottled water (BW) and secondary effluent from the biological treatment plant of the University of Patras, Greece. Table 1 summarizes the main properties of the bottled water and secondary effluent.

### 2.2. Catalytic Experiments

Experiments were conducted in a double-walled cylindrical glass vessel connected with a water bath (model LVF6, Grant instruments, Cambridgeshire, United Kingdom) to control the temperature of the solution at 25 °C. The volume of the reactor was 300 mL. Some experiments were also conducted with the simultaneous use of a Branson ultrasound generator (450 W sonifier) at the fixed frequency of 20 kHz and 36 W/L ultrasound power, as described elsewhere [25].

In a typical run, 200 mL solution of 500 μg/L Piroxicam dissolved in ultrapure water was introduced into the glass vessel and then added the appropriate amount of Iron (II) sulfate heptahydrate. The concentration of iron did not exceeded 2 mg/L, to comply with the European Urban Waste Water Directive 91/271/EEC. Then, the appropriate amount of sodium persulfate was added. After specific time intervals, samples of 1.2 mL were withdrawn from the reactor, quenched with an excess of methanol, filtered with a 0.22 μm PVDF filter, and analyzed using high-pressure liquid chromatography. All of the experiments were run in duplicate, and mean values are quoted as results with a standard error not exceeding 5%.

### 2.3. Estimation of Synergy (S)

Assuming that the decomposition of PIR follows pseudo-first-order kinetics, a standard assumption in similar studies, the change of PIR concentration is described using the following equation:

(3) $$\frac{d[\mathrm{PIR}]}{\mathrm{dt}}=–k_{\mathrm{app}}[\mathrm{PIR}]\;$$

Alternatively, in linearized form:

(4) $$\ln\frac{\left[\mathrm{PIR}\right]}{{\left[\mathrm{PIR}\right]}_{0}}=‒k_{\mathrm{app}}t\;$$

where k_app_ denotes the apparent kinetic constant.

In recent years, in an attempt to increase the efficiency of sewage treatment technologies, the simultaneous use of more than one process has been constantly gaining ground. One way to quantify the interaction between two different processes (Process 1 and Process 2) is the degree of Synergy (S), defined as [27]:

(5) $$S=\frac{k_{\mathrm{Process}1/\mathrm{Process}2}‒k_{\mathrm{Process}1}‒k_{\mathrm{Process}2}}{k_{\mathrm{Process}1/\mathrm{Process}2}}\;$$

where k_Process1/Process2_ is the observed kinetic constant from the simultaneous implementation of the two processes

(6) $$\mathrm{where}\;S\left\{\begin{array}{l} >\;0\;\mathrm{synergistic}\;\mathrm{effect}\\ =\;0\;\mathrm{cumulative}\;\mathrm{effect}\\ <\;0\;\mathrm{antagonistic}\;\mathrm{effect} \end{array}\right\}\;$$

### 2.4. High-Pressure Liquid Chromatography and Total Organic Carbon

Waters Alliance (model 2685) was used for the chromatographic analysis. The system was connected with a kinetex C18 (50 × 2.1 mm and 2.6 μm) column (Phenomenex) that was protected from an in-line stainless steel filter of 0.45 μm (Phenomenex). The flow was 0.2 mL/min with 25:75 acetonitrile and 0.1% phosphoric acid. The detector that was used to monitor the absorbance of Piroxicam at 350 nm was a photodiode array (model 2996, Waters, Milford, MA, USA) [26].

Chemical oxygen demand (COD) was measured according to standard methods using kits from Hach, and total organic carbon (TOC) was measured using an Aurora 1030 W carbon analyzer.

### 2.5. Toxicity

Luminescent marine bacteria *Vibrio fischeri* were used to assess the acute ecotoxicity of Piroxicam prior to and after the proposed treatment. The inhibition of bioluminescence of *V. fischeri* exposed to Piroxicam spiked with secondary effluent for 15 min was measured using a LUMIStox analyzer (Dr Lange, Germany), and the results were compared to an aqueous control [28].

## 3. Results

### 3.1. The Effect of Persulfate

**Figure 1:**
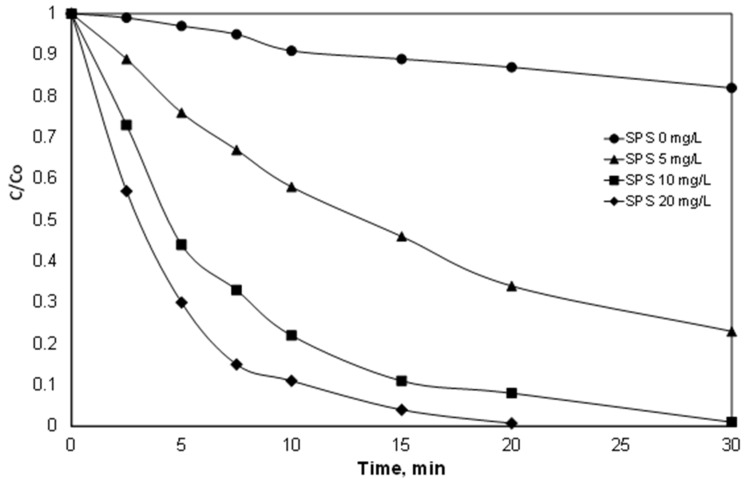
Effect of sodium persulfate on the degradation of 500 μg/L PIR. [Fe^2+^] = 2 mg/L, ultrapure water and inherent pH.

In the first set of experiments, the effect of persulfate dosing was investigated, and the results are shown in Figure 1. It is worth noting that a small decrease in the concentration of PIR was observed even in the absence of the oxidant. Similar results were observed in a previous study from our group on the removal of the endocrine disruptor 17α ethynylestradiol (EE2) using iron, peroxide and simulated solar or ultraviolet radiation [29]. The degradation of the EE2 in the absence of the peroxide was attributed to the reaction of the iron with the dissolved oxygen for the production of the reactive oxygen species according to the following reactions [30]:

(7) $${\mathrm{Fe}}^{2+}+O_{2}\to {\mathrm{Fe}}^{3+}+O_{2}^{•‒}\;$$

(8) $${\mathrm{Fe}}^{2+}+O_{2}^{•-}+2H^{+}\to {\mathrm{Fe}}^{3+}+H_{2}O_{2}\;$$

However, the addition of persulfate increases the concentration of sulfate radicals according to the reaction [21,22,23,24]:

(9) $${\mathrm{Fe}}^{2+}+S_{2}O_{8}^{2‒}\to {\mathrm{SO}}_{4}^{•‒}+{\mathrm{SO}}_{4}^{2‒}+{\mathrm{Fe}}^{3+}\;$$

As shown in Figure 1, the role of the persulfate is crucial. The removal of PIR reached almost 100% after 20 min of reaction with the addition of 20 mg/L of SPS, while for the same time, the PIR removal was only 66% for [SPS] = 5 mg/L. It is worth mentioning that the consumption of 20 mg/L SPS reached 37% after 20 min of reaction.

These results are in agreement with the work of Wei et al. [31], who studied the oxidation of the herbicide bentazone. The three-fold increase in persulfate concentration resulted in a significant increase in the apparent kinetic constant from 0.0357 min^−1^ to 0.0753 min^−1^. In the work of Temiz et al. [32], the degradation of the surfactant Triton X-45 increased from 65% to 88% with an increase in persulfate from 1 to 2.5 mM, using 1 g/L of zero valent iron as the catalyst. Indeed, in a recent study by Metheniti et al. [27], the degradation of the endocrine disrupter propyl paraben by iron-containing magnetic carbon xerogel and persulfate salts was investigated. The researchers observed a significant increase in the kinetic constant when the concentration of persulfate increased. For example, the observed constants for the degradation of 420 μg/L Propyl Paraben with 63 mg/L catalyst at pH 3 were 20.1 × 10^−3^ and 68.6 × 10^−3^ for 25 and 750 mg/L persulfate, respectively.

However, the use of high persulfate loading significantly increases the treatment cost. Also, the final product of persulfate is sulfate ions, which in high concentrations can be considered pollutants. The WHO has suggested that above the threshold of 500 mg/L, people start complaining about the taste of drinking water [33]. In this light, and since optimization was not the goal of this work, an SPS concentration of 20 mg/L was selected for the continuation of this study.

### 3.2. Effect of PIR Initial Concentration

**Figure 2:**
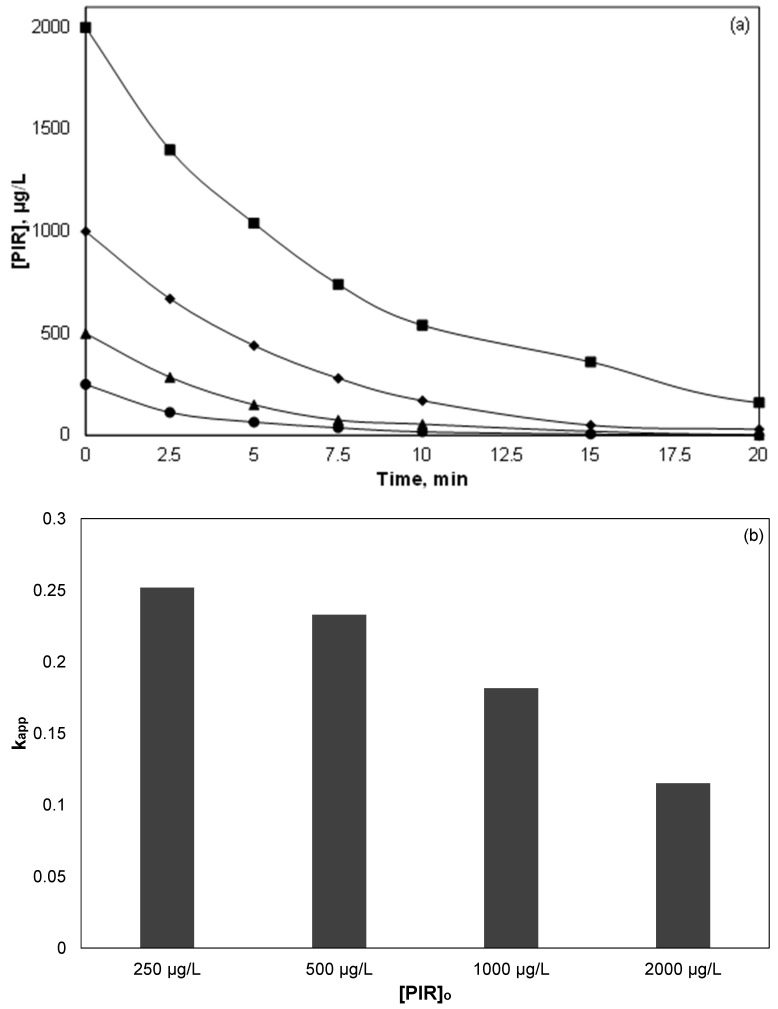
Effect of PIR initial concentration on its decomposition: (**a**) PIR concentration versus time; (**b**) apparent kinetic constant. [Fe^2+^] = 2 mg/L, [SPS] = 20 mg/L, ultrapure water and inherent pH.

Since NSAIDs are detected in different concentrations and mixtures in environmental samples, it is vital to investigate the effect of their initial concentration on their decomposition. Figure 2 shows the destruction of four different initial concentrations of PIR, namely 0.25, 0.5, 1 and 2 mg/L, while all other experimental conditions remain constant. Concentrations of PIR were selected due to the limitations of the analytical methods used in this work. As shown in Figure 2a for a larger initial concentration of PIR, more time is required for the complete degradation of the drug. At the same time, when the initial concentration was 2 mg/L, residual concentration of PIR was detected after 20 min of oxidation; thus, the process did not completely degrade PIR, at least under the conditions in question.

Figure 2b shows the apparent kinetic constants calculated from the normalized concentration data in Figure 2a. It is evident that the kinetic constant decreased as the initial concentration of PIR increased.

In a recent study [34], researchers investigated the treatment of landfill leachates using (industrial) iron activated persulfate. They found that when the initial chemical oxygen demand of the effluent decreased from 121 g/L to 18.5 g/L, the percentage removal increased from 17.2 to almost 48%. Many researchers have studied the dependence of the kinetic constant on the initial concentration of the organics in various processes such as photocatalysis, sonolysis, electrochemical oxidation, and Fenton reaction [4,14]. Most researchers concluded that the oxidation of micropollutants obeys pseudo-first-order kinetics, with a transition from first order to lower order as the initial concentration of the pollutant increasing. Unlikely heterogeneous reactions that the mass transfer is usually the limited step, in homogeneous processes the efficiency is mainly limited due to the low concentrations of reactive oxygen species. Therefore, the rate determining step is the ratio ROS/Pollutant except where the concentration of the oxidant is high enough where self-scavenging may occur.

### 3.3. Effect of pH

**Figure 3:**
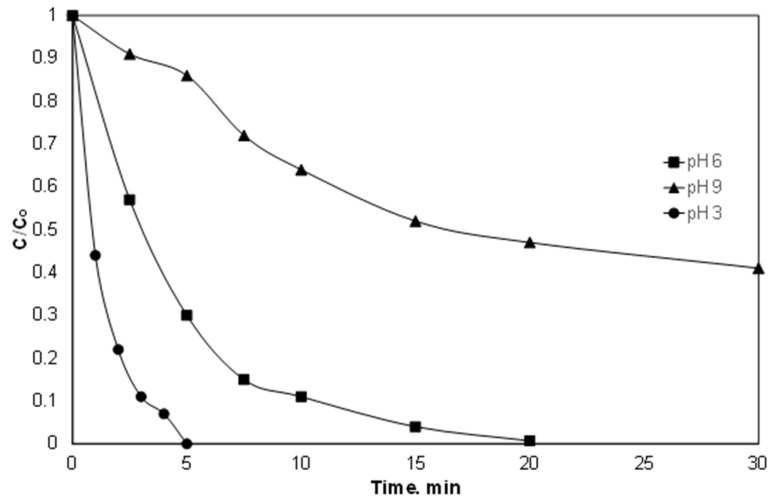
Effect of pH on the degradation of PIR. [PIR] = 500 μg/L, [Fe^2+^] = 2 mg/L, [SPS] = 20 mg/L, ultrapure water and inherent pH.

It is well known that most Fenton and Fenton-like reactions are favored in strongly acidic conditions. This is because iron at higher pH produced sludge due to the precipitation of Fe(OH)_3_ [14]. Indeed, as shown in Figure 3, the superiority of pH 3 versus pH 6.5 and 9 is evident. This is in agreement with the work of Delavaran Shiraz et al. [35], where the removal of catechol using iron activated persulfate was reduced as the pH of the solution increased from 2 to 10.

However, working at pH 3 requires an additional treatment step for the neutralization of effluent, which significantly increases the cost of chemical use and is usually not feasible for large quantities. Therefore, working under “mild” conditions (i.e., pH 6) seems like the golden intersection between process performance, cost and environmental footprint [36].

In addition to iron, pH also affects the distribution of the reactive oxygen species. The sulfate radicals are the dominant species in acidic conditions, while the hydroxyl radicals predominate in the alkaline region [21,22].

(10) $${\mathrm{SO}}_{4}^{‒•}\;+\;{\mathrm{HO}}^{‒}\overset{k\;=\;(4.6–8.3)\;\times \;10^{7}\;M^{‒1}s^{‒1}}{\underset{\mathrm{pH}\;>\;7}{\to }}{\mathrm{SO}}_{4}^{2‒}\;+\;{\mathrm{HO}}^{•}\;$$

**Figure 4:**
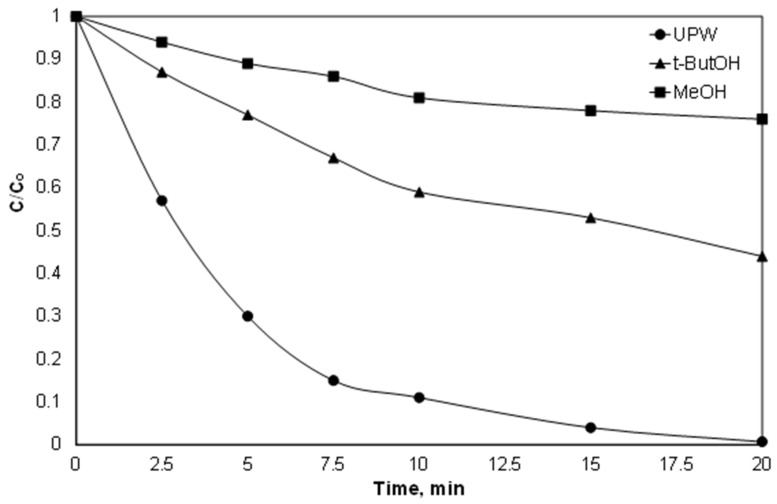
Effect of radical scavengers on the degradation of 1.5 μM PIR at pH 6.5 and ultrapure water with 2 mg/L Fe^2+^, 84 μM SPS and 42 mM of alcohols.

To determine the contribution of both species, additionally, experiments were conducted using t-BuOH and MeOH as radical scavengers. The molar ratio of alcohols and persulfate was 500:1. T-butOH have a much higher kinetic constant for the reaction with hydroxyl radicals than with sulfate radicals [37,38]. On the other hand, the kinetic constants for the reaction of MeOH with both radicals are comparable [37,38]. As shown in Figure 4, the removal of Piroxicam significantly delayed in the presence of alcohols. However, the efficiency of the process was further reduced by the addition of excess of MeOH, thus indicating that both radicals are involved in the removal of PIR, while the sulfate radicals were the dominant species at least in the conditions in question. However, the above-mentioned results should be treated with caution, since they are only accurate for the pH examined (pH 6.5, which decreased to 4.8 during the reaction).

### 3.4. Effect of Bicarbonates and Chlorides

Carbonates are considered one of the most undesirable ions in wastewater, since they scavenge the generated reactive oxygen species according to the following reactions [14]:

(11) $${\mathrm{HO}}^{•}\;+\;{\mathrm{HCO}}_{3}^{‒}\overset{k\;=\;8.6\;\times \;10^{6}{\;M}^{‒1}s^{‒1}}{\to }{\mathrm{CO}}_{3}^{•‒}\;+\;H_{2}O\;$$

(12) $${\mathrm{SO}}_{4}^{•‒}\;+\;{\mathrm{HCO}}_{3}^{‒}\overset{k\;=\;2.6\;\times \;10^{6}{\;M}^{‒1}s^{‒1}}{\to }{\mathrm{HSO}}_{4}^{‒}\;+\;{\mathrm{CO}}_{3}^{•‒}\;$$

**Figure 5:**
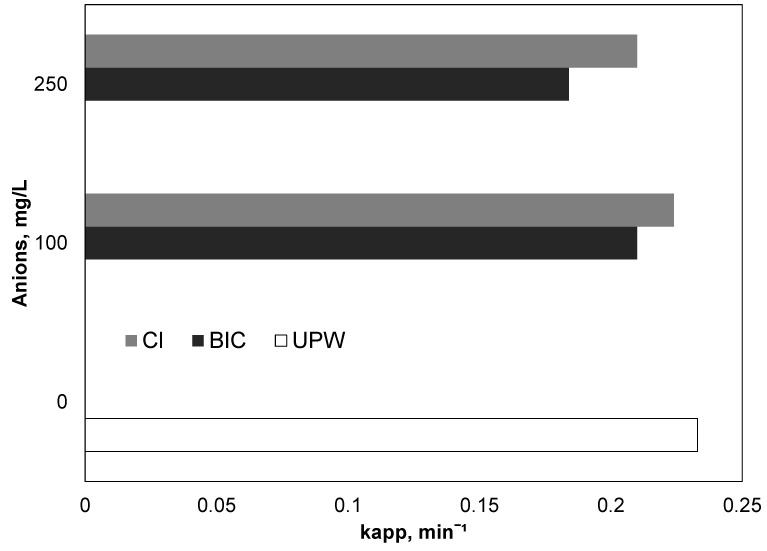
Effect of inorganic ions on the degradation of PIR, [PIR] = 500 μg/L, [Fe^2+^] = 2 mg/L, [SPS] = 20 mg/L, ultrapure water and inherent pH.

Therefore, additional experiments were conducted in the range of 0–250 mg/L of bicarbonates, and the results, expressed as apparent kinetic constants, are depicted in Figure 5. Interestingly, the presence of 250 mg/L of bicarbonate only slightly decreased the observed kinetic constant from 0.233 to 0.184 min^−1^. Similar results were reported by Yang et al. (2014) [39]. The researchers investigated the oxidation of three different organic contaminants namely benzoic acid, 3-cyclohexene-1-carboxylic acid, and cyclohexanecarboxylic acid using UV-activated persulfate. Interestingly, the researchers did not notice any significant difference in the presence or absence of carbonate ion for any of the three pollutants

On the contrary, Nie et al. [40] investigated the destruction of the pharmaceutical Chloramphenicol using iron-activated persulfate. The addition of 1 mM bicarbonates reduced the observed kinetic constant from 0.063 to 0.004 min^−1^. Further growth of bicarbonates up to 100 mM completely inhibited the degradation of the antibiotic. Similar behavior was observed using other oxidants like hydrogen peroxide for the decomposition of emerging contaminants using the solar Fenton process [22]. Thus, the researchers suggested the removal of carbonates and bicarbonates before the Fenton oxidation or the use of higher amounts of iron.

In complex aqueous matrices, the difference in performance is due to the difference in activity of carbonate radicals with the reactive oxygen species such as sulfate and hydroxyl radicals. Although carbonate radicals have a lower potential than other reactive oxygen species, they also have a longer lifetime [41]. Also, it is well known that carbonate radicals are a selective electrophilic reagent with different reactivity towards aromatic compounds. Carbonate radicals as a very strong electron oxidant produce radicals by different targets by both electron transfer and hydrogen abstraction mechanisms [42].

Therefore, the effect of carbonates is not always negative, but is dependent on the experimental conditions, as well as on the target pollutant.

Chloride is another common ion, which is present in environmental samples and wastewater. It is well known that can act as a scavenger for hydroxyl and sulfate radicals according to the reactions [14]:

(13) $${\mathrm{SO}}_{4}^{•‒}\;+\;{\mathrm{Cl}}^{‒}\overset{k\;=\;3\;\times \;10^{8}{\;M}^{‒1}s^{‒1}}{\to }{\mathrm{SO}}_{4}^{2‒}\;+\;{\mathrm{Cl}}^{•}\;$$

(14) $${\mathrm{HO}}^{•}\;+\;{\mathrm{Cl}}^{‒}\overset{k\;=\;4.3\;\times \;10^{9}{\;M}^{‒1}s^{‒1}}{\to }{\mathrm{HO}}^{‒}\;+\;{\mathrm{ClOH}}^{•‒}\;$$

Figure 5 reveals that the effect of chloride at concentrations up to 250 mg/L is insignificant. These results are in line with the work of Metheniti et al. [27], who examined the decomposition of parabens using iron activated persulfate. The research observed a decrease in the kinetic constant from 66 × 10^−3^ to 34.5 × 10^−3^ with the addition of 250 mg/L chlorides. It is worth mentioning that, depending on experimental conditions, there are conflicting results in the literature for the role of chlorides. Rao et al. [43] examined the oxidation of the carbamazepine using Fe^2+^ activated persulfate. They found that, in contrast to other ions such as sulfate, phosphates and nitrates, the removal of carbamazepine significantly increased in the presence of chlorides. The researchers attributed the positive role of chlorides to the generation of Cl^•^ and Cl_2_^•−^ In their pioneer work, Wang et al. [44] examined the influence of chlorine ion in relation to different activators of persulfate and peroxymonosulfate (thermal, UV and Cobalt). They concluded that the effect of chlorides is strictly related to experimental conditions.

Indeed, in previous work, we investigated the photodegradation of the carcinogenic dye methyl orange using UV-activated persulfate [45]. The analysis, which used statistical methods, namely two-level factorial design, revealed that the effect of sodium chloride was not statistically significant in the decolorization of methyl orange in comparison to other factors such as the initial concentration of the pollutant, pH and treatment time.

### 3.5. Effect of Humic Acid

**Figure 6:**
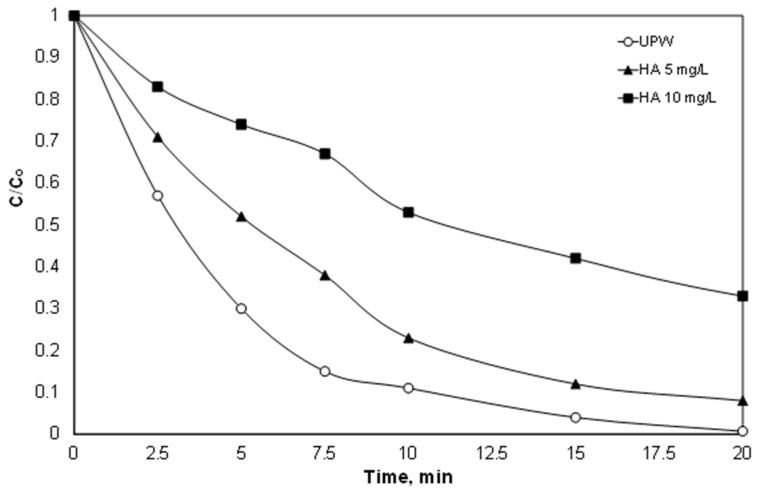
Effect of organic matter (humic acid) on the destruction of PIR. [PIR] = 500 μg/L, [Fe^2+^] = 2 mg/L, [SPS] = 20 mg/L, ultrapure water and inherent pH.

However, in addition to the anions, environmental samples usually also contain natural organic matter (NOM), which in the case of secondary effluent is often called effluent organic matter (efom), and it is well known that it is highly resistant to further oxidation [46]. Therefore, in another set of experiments, the effect of natural organic matter on the degradation of PIR was examined. To simulate the presence of organic matter, experiments were conducted in synthetic solutions containing 5 and 10 mg/L of technical-grade humic acid, and the results are depicted in Figure 6.

Unfortunately, the presence of humic acid significantly reduced the oxidation of PIR. The % removal of PIR was reduced from 69% after 5 min in ultrapure water to 48% and 26% for 5 and 10 mg/L, respectively. In their pioneer work, Ahmed et al. [47] examined the destruction of carbamazepine using the solar /Fe^2+^/SPS process. When the concentration of humic acid increased from 1 to 20 mg/L, the observed kinetic constant decreased by almost half from 0.154 to 0.074 min^−1^. This inhibition is strongly related to the reaction between humic acid and generated reactive oxygen species [47]. Since the radicals are considered to be unselective, they also have high kinetic constants for the reaction with humic acid). In addition, the ratio TOC_HA_/TOC_PIR_ is greater than 9; therefore, PIR is only a small fraction of the carbon contained in the humic acid.

### 3.6. Effect of Water Matrix

**Figure 7:**
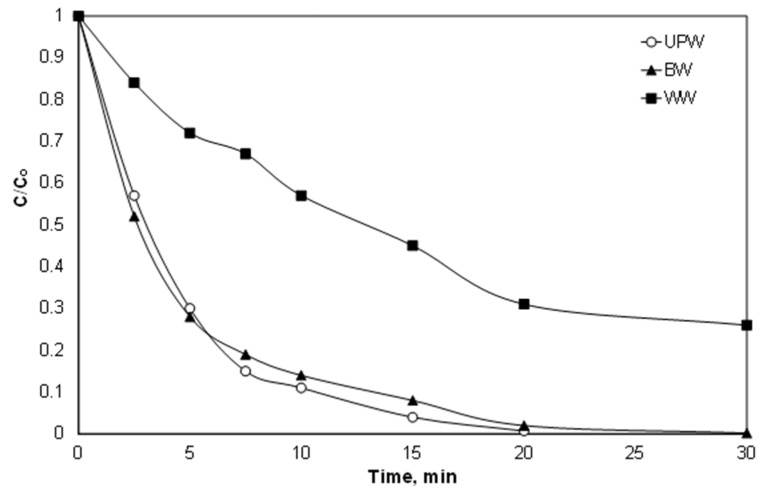
Effect of the water matrix on the oxidation of PIR. [PIR] = 500 μg/L, [Fe^2+^] = 2 mg/L, [SPS] = 20 mg/L, ultrapure water and inherent pH. UPW: ultrapure water BW: bottled water, WW: secondary effluent.

Despite the effect of individual ions and organic matter, from an engineering point of view, the most important is the efficacy of the proposed process in real matrices. Therefore, experiments were performed in environmental matrices like bottled water and secondary treated effluent and the results are shown in Figure 7. The results are in line with the observation about the role of ions like carbonate and chlorides, as well as with the role of organic matter. The observed kinetic constant was only slightly decreased for the experiment performed in bottled water from 0.23 to 0.20 min^−1^, while it decreased significantly for secondary effluent (0.051 min^−1^). At the same time, the COD reduction was 23%, while the total organic carbon (TOC) was reduced by less than 7%, implying only partial degradation of the secondary effluent.

In a recent study by Matthaiou et al. [48], who studied the degradation of the endocrine disrupter propyl paraben using persulfate activated by iron derived from red mud, researchers found that the apparent kinetic constant was almost 4-fold lower in secondary effluent than ultrapure water. It is, therefore, evident that in real and complex aqueous matrices, it is necessary either to use higher concentrations of the (often costly) reagents, or to couple them with some other technology to satisfy strict limits and legislation.

### 3.7. Effect of Ultrasound

To avoid intensive conditions such as the costly use of large quantities of chemicals or catalysts, an alternative way to increase the efficiency of AOPs is the simultaneous application of different AOPs. Under this perspective, we examined the simultaneous use of iron, persulfate, and ultrasound. Ultrasound is another interesting advanced oxidation process that is based on the generation of reactive oxygen species due to the expansion and collapse of cavities under the influence of a sound field [14].

**Figure 8:**
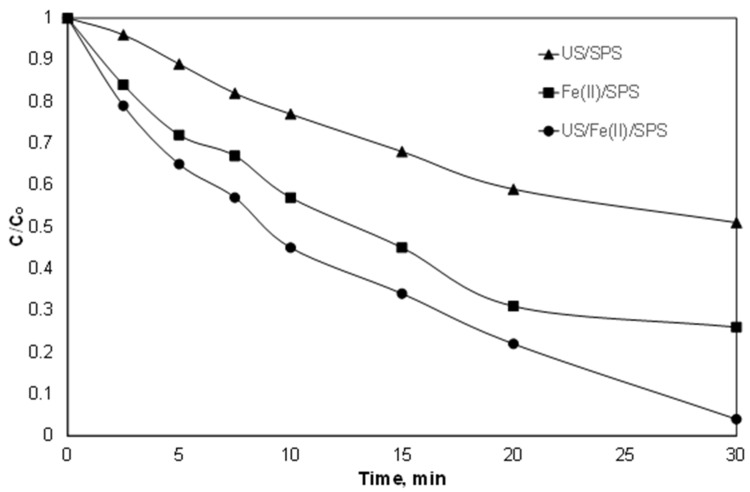
Normalized concentration of PIR degradation with Fe^2+^/SPS, US/SPS and Fe^2+^/US/SPS. [PIR] = 500 μg/L, [Fe^2+^] = 2 mg/L, [SPS] = 20 mg/L, ultrapure water and inherent pH. US: ultrasound.

Sonochemistry is often used in similar studies, since apart from the formation of extra radicals, it also increases the mass transfer between the oxidants, the reactants and the catalysts [35]. As shown in Figure 8, and as already demonstrated [25], low-frequency ultrasound can decompose PIR at high power density, while the presence of SPS can produce additional ROS and to enhance the degradation rate. However, the simultaneous use of Fe^2+^, SPS, and ultrasound increased the observed kinetic constant that was 0.051, 0.024 and 0.091 min^−1^ for Fe^2+^/SPS, US/SPS and Fe^2+^/US/SPS, respectively.

These results are in agreement with the work of Seid-Mohammadi et al. [49], who investigated the Fe^2+^/SPS/US for the decolorization of the carcinogenic dye Acid Blue 113 (AB 113). The authors found that the removal of 50 mg/L after 45 min of treatment, 2.5 mM of S_2_O_8_^2−^, 0.5 mM of ferrous sulfate and pH 3 increased from 80.56% to 94.3% with the simultaneous use of ultrasound at 40 kHz.

One way to quantify the interaction between two different processes is the degree of Synergy (S):

In our case, S was defined as [27,50]:

(15) $$S\;=\;\frac{k_{\mathrm{Fe}/\mathrm{SPS}/\mathrm{US}}‒k_{\mathrm{Fe}/\mathrm{SPS}}‒k_{\mathrm{US}/\mathrm{SPS}}}{k_{\mathrm{Fe}/\mathrm{SPS}/\mathrm{US}}}\;$$

(16) $$S\;=\;1‒\frac{{(k}_{\mathrm{Fe}/\mathrm{SPS}}\;+\;k_{\mathrm{US}/\mathrm{SPS}})}{k_{\mathrm{Fe}/\mathrm{SPS}/\mathrm{US}}}\;$$

Applying Equation (15) for the simultaneous use of Fe^2+^/US/SPS the synergy index was calculated 18.3%. Thus, there is a small synergy between the two processes, and the phenomenon is not cumulative. On the contrary, in a similar study by Metheniti et al. [27], the researchers observed a synergy of up to 50% between the (heterogeneous) iron activated persulfate and the simultaneous use of low-frequency ultrasound. The almost threefold synergy observed is probably due to the large increase in mass transfer by the ultrasound waves in heterogeneous reactions, which is not the case for this work.

### 3.8. Toxicity

**Figure 9:**
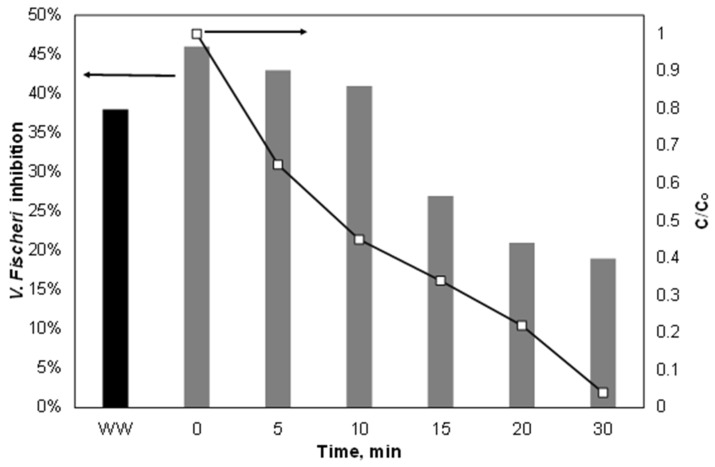
Variation of PIR and toxicity during the Fe^2+^/SPS treatment in secondary effluent. [PIR] = 500 μg/L, [Fe^2+^] = 2 mg/L, [SPS] = 20 mg/L, ultrapure water and inherent pH.

Finally, the variation of ecotoxicity during the treatment of 500 μg/L PIR spiked in the secondary effluent was assessed using the bacterium *Vibrio Fischeri* as an indicator, as well as the combined ultrasound/Fe^2+^/SPS process, and the results are depicted in Figure 9. As shown, the highest inhibition rate observed was due to the water matrix itself excluding PIR, where the latter made only a small contribution to *V. Fischeri* inhibition. The inhibition (which never exceeded 50%) decreased during the treatment; however, it did not do so with the same trend as the removal of PIR. This was due to the generation of byproducts from the oxidation of the organic matter of the secondary effluent that had different inhibition from both the parent compounds and the piroxicam.

Contrary to our results, Temiz et al. (2016) [32] examined the destruction of Triton X-45 (TX-45) using persulfate activated by zero valent iron. Using 1 g/L ZVI; 2.5 mg PS and pH 5 for the treatment of 20 mg/L TX-45, the acute toxicity expressed as *V. fischeri* inhibition decreased slowly from 69% to 23% after 40 min of treatment and remained almost stable in the range 21–26%. It is worth noting that the profile of toxicity followed (in parallel) the profile of TX-45 degradation. However, these experiments were performed in distilled water spiked with TX-45; therefore, the byproducts were derived only from the oxidation of TX-45, without considering the effect of the actual aqueous matrix on the toxicity.

On the other hand, Michael-Kordatou et al. [51] observed a temporary increase in phytotoxicity (expressed as shoot growth inhibition and root growth inhibition) in the first 30 min, followed by a further decrease during the UVC/persulfate treatment of secondary effluent spiked with 100 μg/L of erythromycin. The authors have attributed this behavior to the formation of new organic entities with phytotoxic effects. Therefore, proper evaluation for any process efficiency requires the combination of chemical and biological indicators.

## 4. Conclusions

This work investigated the degradation of the NSAID piroxicam using iron-activated persulfate with a particular emphasis on operating conditions and water matrix. The conclusions derived from this work can be summarized as follows:

- The role of SPS was vital, and even a small dose (20 mg/L) could degrade 500 μg/L of Piroxicam with 2 mg/L Fe^2+^ in less than 20 min at natural pH.
- The role of pH was crucial, but working under “mild” conditions (i.e., pH 6–6.5) seems like the golden intersection between process performance, cost and environmental footprint.
- The effect of inorganic ions in environmentally relevant concentrations was almost negligible. On the contrary, the presence of organic matter inhibited the degradation of PIR.
- The simultaneous use of ultrasound showed only a small synergy between low-frequency ultrasound and iron-activated persulfate.
- The variation in toxicity in secondary effluent did not follow PIR removal possibly due to the generation of toxic byproducts.
- Further research is needed to optimize the process and investigate efficiency under real conditions.

## Figures and Tables

**Table 1:** Characteristics of the water matrices.

Property	Ultrapure Water (UPW)	Bottled Water (BW)	Wastewater Effluent (WW)
pH	6.3	7.2	8.2
Conductivity, μS/cm	0.062	407	817
TOC, mg/L			4.6
Bicarbonate, mg/L		198	191
Chloride, mg/L		7.4	72
Sulfate, mg/L		13	26
Nitrate, mg/L		2.1	4.7
